# Mycosporine-like Amino Acids Biosynthesis in *Asterarcys* sp. Driving by Phosphorus Limitation: Evidence from Physiological and Transcriptomic Analyses

**DOI:** 10.3390/md24050161

**Published:** 2026-04-30

**Authors:** Liang Wei, Hualian Wu, Jiayi Wu, Houbo Wu, Jinting Lv, Tao Li, Wenzhou Xiang

**Affiliations:** 1 State Key Laboratory of Breeding Biotechnology and Sustainable Aquaculture, Guangdong Key Laboratory of Marine Materia Medica, South China Sea Institute of Oceanology, Chinese Academy of Sciences, Guangzhou 510301, China; weiliang23@mails.ucas.ac.cn (L.W.); hlwu@scsio.ac.cn (H.W.); wuhoubo@scsio.ac.cn (H.W.); lv_jt2017@126.com (J.L.); 2 University of Chinese Academy of Sciences, Beijing 101408, China; 3 Greater Bay Area Institute of Precision Medicine (Guangzhou), Fudan University, Guangzhou 510301, China; kayeewu@scsio.ac.cn

**Keywords:** MAAs, *Asterarcys*, phosphorus limitation, transcriptomic

## Abstract

Mycosporine-like amino acids (MAAs), a class of secondary metabolites characterized by a cyclohexenone or cyclohexenimine ring structure bound to amino acid residues, are widely distributed in algae. These compounds exhibit strong ultraviolet-absorbing and antioxidant activities, making them attractive candidates for natural sunscreen formulations. However, the low productivity of MAAs in microalgae severely hampers commercial viability. *Asterarcys* sp., a fast-growing, heat- and light-tolerant microalga, has recently been demonstrated to produce high levels of MAAs under UV irradiation. In this study, phosphorus limitation was found to stimulate rapid MAAs accumulation in *Asterarcys* sp. SCSIO-46548. After eight days of cultivation, microalgal cells grown in phosphorus-free medium (0 mg L^−1^) showed a sixfold higher MAAs content (1.08% DW) compared to the group supplied with 5.60 mg L^−1^ phosphorus (0.18% DW). However, the accumulation of MAAs began to plateau under phosphorus deprivation. Based on integrated homology alignment with cyanobacteria and functional domain validation, a putative biosynthetic pathway for mycosporine-serine in *Asterarcys* sp. SCSIO-46548 was proposed. Importantly, the gene expression of desmethyl-4-deoxygadusol synthase (DDGS) exhibited a 2.75-fold upregulation under phosphorus limitation. Complementary bioinformatic analyses further characterized the subcellular localization and major physicochemical properties of the candidate enzymes involved. In conclusion, phosphorus limitation is an effective strategy to enhance MAAs production in *Asterarcys* sp. SCSIO-46548 by upregulating the expression of key biosynthetic genes, such as DDGS. This finding provides an effective solution to the low MAAs productivity in microalgae cultivation.

## 1. Introduction

With the steady increase in the incidence of UV-induced skin cancer worldwide in recent decades [1,2], public awareness of sun protection and the demand for highly effective sunscreen products have grown significantly. Conventional commercial sunscreens rely on synthetically produced organic ultraviolet filters, such as anthranilates, dibenzoylmethanes, para-aminobenzoic acid (PABA) [3]. However, the efficacy and safety of these compounds are limited by their tendencies for photodegradation and potential ecological toxicity [4]. Biosynthesized sunscreen agents with enhanced safety and reliability are attracting interest in consumer markets.

Mycosporine-like amino acids (MAAs) are a class of secondary metabolites produced by aquatic organisms as a protective response to ultraviolet (UV) radiation and environmental stress [5,6]. These compounds are characterized by a cyclohexenone or cyclohexenimine ring bound to amino acid residues or their derivatives, a structure which underpins their strong UV-absorbing and antioxidant capacities [7]. Capitalizing on these dual photoprotective and antioxidant functions, MAAs have transitioned from laboratory research to commercial cosmetic ingredients. Notable examples include Helioguard™ 365 (Mibelle Biochemistry Group) and HELINORI^®^ [8,9], both derived from macroalgae and rich in MAAs such as shinorine and porphyra-334. Consequently, sunscreen products utilizing MAAs as the core functional ingredient possess strong commercial appeal and substantial market prospects.

*Asterarcys*, a green alga within the family of Scenedesmaceae, has the characteristics of rapid growth, high temperature resistance, strong light resistance, and richness in α-linolenic acid and hexadecenol bioactive substances [10,11]. Recently, *Asterarcys* sp. SCSIO-46548. was found to produce high levels of MAAs under ultraviolet (UV) radiation [12]. Further analysis of the MAAs in *Asterarcys* sp. SCSIO-46548 revealed a primary constituent with a molecular mass of 275.1078 Da, *m*/*z* = 276.1069 (Figures S1–S3). The characteristic fragment ions at *m*/*z* 258, 228, 212, 199, and 182 (Figure S4) align with mycosporine-serine [13,14]. Therefore, *Asterarcys* may be an ideal candidate strain for large-scale cultivation of MAAs.

**Figure 1:**
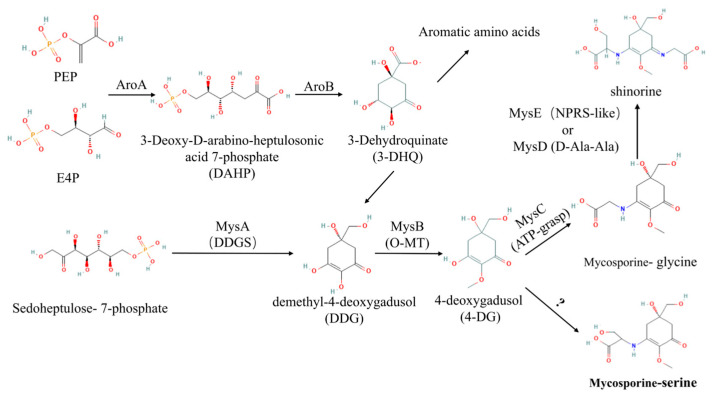
The biosynthetic pathway of MAAs in microalgae [15].

Mycosporine-like amino acids are known to be biosynthesized through two main metabolic pathways (Figure 1)—the shikimate pathway and the pentose phosphate pathway [15]. The shikimate pathway generates 3-dehydroquinate (3-DHQ) from phosphoenolpyruvate (PEP) and erythrose 4-phosphate (E4P), catalyzed by enzymes including 3-deoxy-D-arabino-heptulosonate 7-phosphate synthase (AroA2) and 3-DHQ synthase (AroB). 3-DHQ is subsequently converted to demethylated 4-deoxygadusol (DDG). Alternatively, DDG is directly formed from sedoheptulose 7-phosphate via the pentose phosphate pathway by the enzyme DDG synthase (DDGS). DDG serves as the common intermediate where the shikimate and pentose phosphate pathways converge. It is subsequently methylated by O-methyltransferase (O-MT) to form 4-deoxygadusol (4-DG). 4-DG is the core precursor for all MAAs. Mycosporine-glycine (M-Gly) is synthesized when MysC ligates glycine to 4-DG; the subsequent addition of serine to M-Gly by either D-alanine D-alanine ligase homolog (Ddl) or non-ribosomal peptide synthetase-like enzyme (NRPS-like) then yields shinorine. Notably, these two pathways are not mutually exclusive and have been shown to coexist in microalgae. Geraldes et al. [16] employed genomic analysis to identify the concurrent presence of genes encoding key enzymes from both the shikimate and pentose phosphate pathways in *Sphaerospermopsis torques-reginae*, and further demonstrated through inhibitor assays that the shikimate pathway plays a predominant role in MAAs biosynthesis. Similarly, Mogany et al. [17] identified a unique six-gene mys cluster in *Euhalothece* sp. encoding homologs of both DHQ synthase and DDGS, thereby providing additional genetic evidence for dual-pathway MAAs biosynthetic capacity in cyanobacteria. Nevertheless, mycosporine-serine has not been previously reported in microalgae, and its biosynthetic pathway remains uncharacterized.

Current research on the induction of MAAs biosynthesis in microalgae has primarily focused on environmental stressors such as UV and salinity stress. Boucar et al. [18] found that UV-B radiation significantly enhances the synthesis of palythine-serine and carotenoids in the cyanobacterium *Pseudanabaena* sp. Furthermore, Tsintzou et al. [19] demonstrated that the green microalga *Jaagichlorella luteoviridis* grown under UV, salinity, or heat stress exhibited a marked increase in MAAs content, and transcriptomic analysis revealed significant upregulation of ArioC and AroM/Aro1 SAM methyltransferases. However, the implementation of such stress conditions in large-scale cultivation systems entails considerable economic costs and operational challenges, limiting their direct applicability to industrial production. Accordingly, there is a critical imperative to develop cost-effective and industrially scalable strategies for boosting MAAs accumulation in microalgal cultivation systems.

Phosphorus (P) serves as one of the most critical nutrients for microalgal growth, playing a fundamental role in synthesizing essential cellular components such as phospholipids, nucleotides, and the cellular energy currency ATP [20,21]. Phosphorus limitation induces physiological changes, notably reduced chlorophyll synthesis and increased photo-oxidative stress, which probably promotes the accumulation of MAAs [22,23]. An inverse correlation between phosphorus availability and the accumulation of MAAs has been reported in *Glenodinium foliaceum* [24]. In contrast, elevated phosphate levels in the culture mediums have been shown to enhance the yield of MAAs in *Fischerella* sp. F5 [25]. This apparent discrepancy may be attributed to the species-dependent phosphorus metabolic capacity among different microalgae.

The study investigated the effects of phosphorus concentration on growth, photosynthetic performance, the synthesis of MAAs and the biochemical composition of *Asterarcys* sp. SCSIO-46548. Furthermore, key enzymes implicated in the MAAs biosynthetic pathway were identified through homology-based alignment and functional domain analysis. The transcriptional responses to phosphorus limitation were further elucidated by transcriptomic profiling. Additionally, bioinformatic tools were employed to predict the subcellular localization and fundamental physicochemical properties of these candidate enzymes. The findings elucidate a nutrient-based strategy for effectively inducing MAAs accumulation under industrial cultivation conditions and provide guidance for future genetic engineering aimed at enhancing MAAs production.

## 2. Results and Discussion

### 2.1. Growth Response of Asterarcys sp. SCSIO-46548 to Different Phosphorus Concentrations

**Figure 2:**
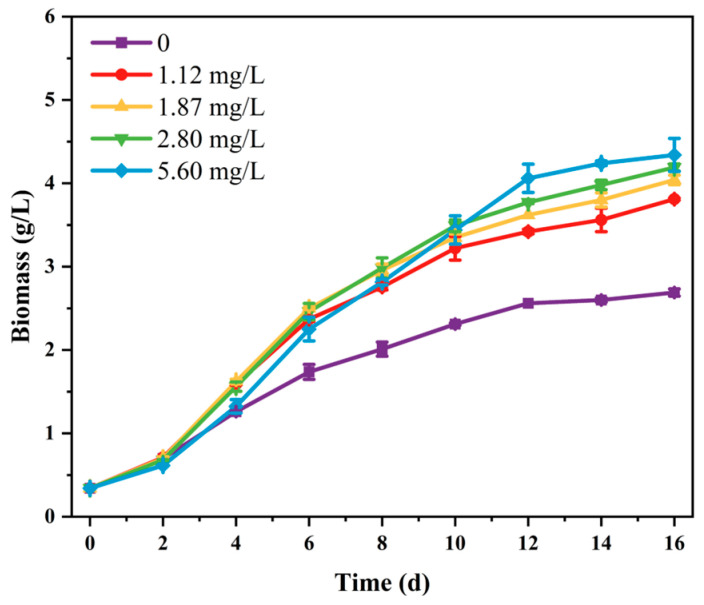
The growth of *Asterarcys* sp. SCSIO-46548 in different phosphorus concentrations.

Phosphorus is an essential macronutrient that plays fundamental roles in the synthesis of key cellular components and signal transduction pathways [26,27]. In this study, SCSIO-46548 was cultured under different phosphorus concentrations, which were regulated by varying K_2_HPO_4_·3H_2_O levels (0, 8, 13.3, 20, and 40 mg/L), corresponding to initial phosphorus concentrations of 0, 1.12, 1.87, 2.80, and 5.60 mg/L. As shown in Figure 2, the biomass of SCSIO-46548 showed an increasing trend during cultivation. By the end of the cultivation, the treatment with an initial phosphorus concentration of 5.60 mg/L yielded the maximum biomass (4.34 g/L), which was 1.61 times that of the phosphorus-free group (2.69 g/L). However, the biomass at 5.60 mg/L phosphorus was initially low during the early stage of cultivation. This could be related to the inhibitory effect of excess phosphorus on the growth of microalgal cells [28].

### 2.2. Photosynthetic Performance of Asterarcys sp. SCSIO-46548 Under Different Phosphorus Concentration

**Figure 3:**
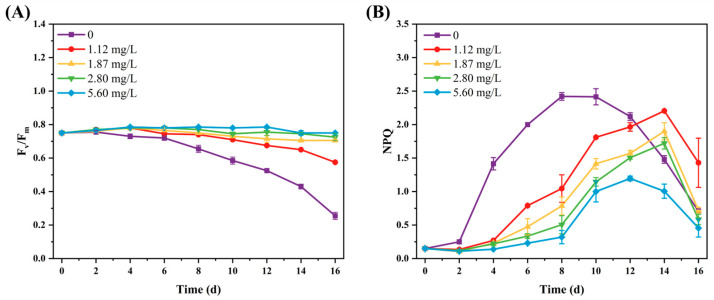
The photosynthetic parameters of *Asterarcys* sp. SCSIO-46548 under variable phosphorus concentration. (**A**) F_v_/F_m_; (**B**) NPQ.

The effects of different phosphorus concentrations on the photosynthetic parameters of SCSIO-46548 are shown in Figure 3. When the initial phosphorus concentration exceeded 1.87 mg/L, the F_v_/F_m_ of microalgae cells remained stable between 0.70 and 0.80. In contrast, at concentrations below 1.12 mg/L, the F_v_/F_m_ showed a time-dependent decline. On day 16 of the cultivation, the F_v_/F_m_ of microalgae cells in the 0 mg/L phosphorus group had decreased to 0.26, which was merely 34% of that in the 5.60 mg/L phosphorus group (0.75). This result indicated a reduction in the primary light-energy conversion efficiency of PS II [29]. Non-photochemical quenching (NPQ) displayed a consistent rise-and-fall pattern across the phosphorus gradient. An inverse relationship between NPQ levels and phosphorus concentration was observed within the first 10 days. In the later stage of cultivation, the NPQ in the low-phosphorus groups began to decline rapidly from day 10 to day 16, which might be associated with damage to the photosynthetic system. This pattern suggested that phosphorus limitation could induce photo-oxidative stress, whereas severe deprivation is likely to cause damage to the photosynthetic system.

### 2.3. The Biosynthesis of MAAs in Asterarcys sp. SCSIO-46548 Under Different Phosphorus Concentration

**Figure 4:**
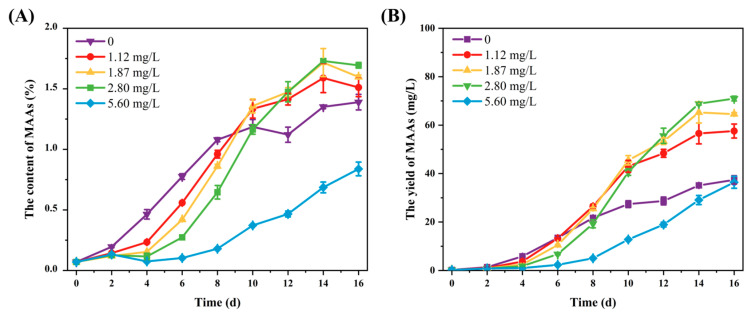
The MAAs accumulation of *Asterarcys* sp. SCSIO-46548 under different phosphorus concentration. (**A**) The content of MAAs; (**B**) the yield of MAAs.

The content of MAAs in SCSIO-46548 increased gradually over the cultivation period (Figure 4). During day 0 to day 8, the accumulation of MAAs showed a negative correlation with phosphorus concentration. On day 8 of cultivation, the MAAs content in the phosphorus-free group (0 mg/L) reached 1.08% DW, which was six times higher than that of the 5.60 mg/L phosphorus group (0.18% DW). These results demonstrated that the biosynthesis of MAAs in SCSIO-46548 was promoted by phosphorus limitation. On day 10, the group with a phosphorus concentration of 1.87 mg/L exhibited the highest MAAs content (1.36% DW). Subsequently, during day 12 to day 16, the peak accumulation shifted to the group with 2.80 mg/L phosphorus, reaching a maximum value of 1.73% DW. In contrast, the control group (5.60 mg/L) consistently displayed the lowest MAAs content throughout the cultivation period. This dynamic, non-linear pattern of MAAs accumulation in response to phosphorus concentration and time aligned with the progressive depletion of phosphorus in the culture medium. The synthesis of MAAs was induced within a specific range of phosphorus limitation, whereas severe phosphorus deprivation led to a decline in the accumulation rate. The observed phenomenon can be attributed to a decrease in chlorophyll content [30], which subjected algal cells to photo-oxidative stress, thereby inducing the synthesis of MAAs as a compensatory photoprotective mechanism. Additionally, the decrease in MAAs content induced by phosphorus deprivation may stem from cell death.

### 2.4. Biochemical Composition in Response to Phosphorus Availability

**Figure 5:**
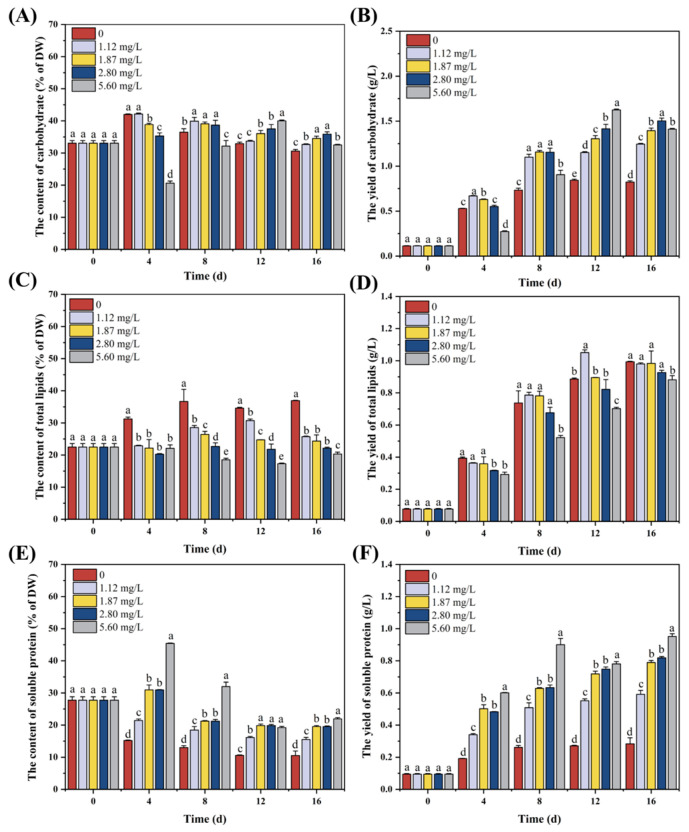
Biochemical composition of *Asterarcys* sp. SCSIO-46548 under different phosphorus concentration. (**A**) Carbohydrate content; (**B**) total lipid content; (**C**) soluble protein content; (**D**) carbohydrate productivity; (**E**) total lipid productivity; (**F**) soluble protein productivity. Different letters indicate significant differences between groups (*p* < 0.05, LSD test).

The determination of biochemical composition, particularly protein and carbohydrate content, was essential for understanding the nitrogen and carbon flux associated with MAAs synthesis, as MAAs are nitrogenous compounds synthesized from precursors derived from the pentose phosphate pathway [31]. Except for the initial phosphorus concentration of 5.60 mg/L, the carbohydrate content of SCSIO-46548 in all other groups exhibited an initial increase followed by a decrease over the cultivation period (Figure 5A). The carbohydrate content on day 4 showed a negative correlation with the initial phosphorus concentration, whereas this relationship shifted to a positive correlation by day 12. These phenomena can be attributed to prolonged phosphorus deprivation, which induces both cell death and a metabolic shift in carbon allocation from polysaccharides to lipids [32]. As cultivation progressed and phosphorus was gradually depleted, microalgal cells from groups with different initial phosphorus concentrations entered a state of phosphorus limitation sequentially over time. This sequential onset of stress ultimately resulted in a positive correlation between carbohydrate content and the initial phosphorus concentration during the final stages of cultivation. On day 16 of cultivation, SCSIO-46548 with 2.80 mg/L of phosphorus showed the highest carbohydrate production, reaching a content of 35.87% DW and a yield of 1.50 g/L, respectively.

As shown in Figure 5B, a negative correlation was observed between initial phosphorus concentration and total lipid content during the cultivation, indicating that phosphorus limitation promoted lipid accumulation in SCSIO-46548. On day 16, microalgal cells cultured at 0 mg/L phosphorus exhibited the highest total lipid content of 36.94% DW, which represented an 81.88% increase over the 5.60 mg/L phosphorus group (20.31% DW). However, the lipid yield showed no significant difference in the later cultivation stage due to growth suppression caused by phosphorus limitation.

In contrast to total lipids, the soluble protein content in SCSIO-46548 exhibited a positive correlation with the initial phosphorus concentration (Figure 5C). When the initial phosphorus concentration was above 1.87 mg/L, soluble protein content showed an initial increase followed by a decrease over time. Conversely, at concentrations below 1.12 mg/L, it declined progressively throughout the cultivation. This pattern suggests that phosphorus limitation suppressed soluble protein synthesis. This metabolic reprogramming was triggered when phosphorus availability fell below a critical threshold between 1.12 and 1.87 mg/L. On day 16 of cultivation, SCSIO-46548 under 5.60 mg/L phosphorus achieved the highest soluble protein content (21.93% DW) and yield (0.95 g/L).

In summary, phosphorus limitation induced significant metabolic reprogramming in SCSIO-46548, redirecting carbon and nitrogen fluxes from protein synthesis toward the accumulation of energy storage compounds, primarily carbohydrates and lipids. Under phosphorus limitation, the degradation of proteins likely provided carbon skeletons and energy to support the synthesis of polysaccharides and lipids [33,34]. As phosphorus deprivation became more severe or prolonged, a further metabolic adjustment occurred, channeling carbon flow from polysaccharide synthesis toward lipid accumulation. This strategy may represent a more efficient adaptive response to extreme stress, as lipids offer a higher energy density and greater cellular stability under unfavorable conditions [35,36]. Similar patterns have been reported in other microalgae, where severe phosphorus deprivation triggered the upregulation of key genes involved in fatty acid biosynthesis (e.g., DGAT) while suppressing starch synthesis [37].

### 2.5. Expression Level of Several Key Enzymes Related to MAAs Synthesis Under Phosphorus Limitation

To elucidate the mechanisms of MAAs accumulation under phosphorus limitation, transcriptome analysis was performed on microalgal cells cultured at total phosphorus concentrations of 1.87 mg/L (the phosphorus-limited group) and 5.60 mg/L (the control group) on day 8. This group was chosen to avoid the confounding effects of extreme phosphorus deprivation while still eliciting significant MAAs production for mechanistic analysis.

A total of 38,733 genes were obtained through RNA extraction and assembly, of which 18,749 sequences were successfully functionally annotated, yielding an annotation rate of 48.41% (Table S1). Among the various databases, the NR database yielded the highest number of annotated sequences, with 17,555 sequences (45.32% of all genes) showing homology to known sequences. Of these, 11,606 unigenes exhibited significant homology (E-value < 1.0 × 10^−30^) to known genes in the NR database. Annotations from other databases were as follows: 14,401 sequences (37.18%) in the GO database, 12,674 sequences (32.72%) in the Pfam database, 12,180 sequences (31.45%) in the eggNOG database, 10,453 sequences (26.99%) in the Swiss-Prot database, and 8414 sequences (21.72%) in the KEGG database.

**Figure 6:**
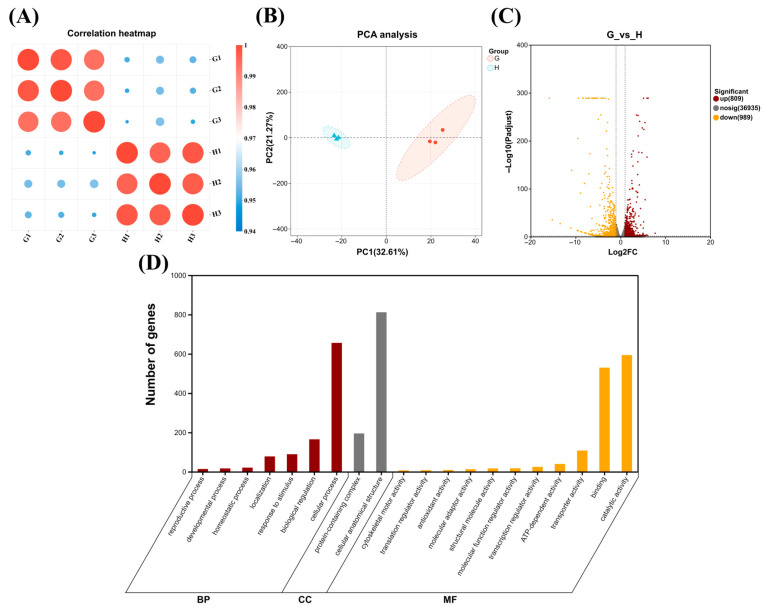
Sample correlation heatmap (**A**); PCA (**B**); volcano plots of DEMs (**C**); GO classification of differentially expressed genes (**D**); groups: G, phosphorus-limited; H, the control.

Principal component analysis (PCA) demonstrated high reproducibility across biological replicates, with intra-group coefficients of determination (R^2^) ranging from 0.99 to 1.00 (Figure 6A). Samples from the two conditions formed distinct, well-separated clusters in the PCA score plot, confirming significant inter-group differences (Figure 6B). Differential expression analysis, using thresholds of an adjusted *p*-value < 0.05 and |log_2_FC| ≥ 1, identified 1798 significantly differentially expressed genes (DEGs) (Figure 6C). Compared to the control, phosphorus-limited cells exhibited 809 upregulated and 989 downregulated genes. Gene Ontology (GO) enrichment analysis of these DEGs revealed their functional distribution (top 20 terms shown in Figure 6D). Within biological processes (BP), DEGs were predominantly associated with cellular processes and biological regulation. For molecular function (MF), significant enrichment was observed in binding and catalytic activity. Regarding cellular components (CP), the main enrichments were cellular anatomical entity and protein-containing complex.

#### 2.5.1. Photosystem

As essential photoprotective agents, the biosynthesis of MAAs is closely linked to the state and function of the photosynthetic system in microalgae [38,39]. As shown in Table 1, phosphorus limitation resulted in the coordinated downregulation of the expression levels of genes encoding key light-harvesting proteins (LHca2, LHca4, LHcb1, and LHcb2) and PSI-binding subunits (PsaG and PsaO). This might have represented a protective feedback mechanism, where cells reduced excess light absorption to prevent photo-oxidative damage to the photosystem. At the electron transfer and energy conversion stage, the downregulation of key electron transport proteins at the expression level, including plastocyanin ferredoxin-NADP+ reductase (PetH), cytochrome c6 (PetJ), and PSII oxygen-evolving complex subunits (PsbP and PsbQ), led to the inhibition of electron flow between the two photosystems and suppression of NADPH synthesis. Furthermore, a marked decrease was observed in the expression level of the ATP synthase gamma subunit, indicating that the efficiency of converting the proton-motive force into chemical energy was reduced in SCSIO-46548 under phosphorus limitation [40]. Therefore, phosphorus limitation not only suppressed light-harvesting but also impaired the entire energy conversion chain in SCSIO-46548, which might have indirectly enhanced the biosynthesis of MAAs as a photoprotective compensation mechanism.

#### 2.5.2. The Biosynthesis of MAAs

The characteristic fragment ions of primary MAAs in SCSIO-46548 at *m*/*z* 258, 228, 212, 199, and 182 (Figures S1–S4) align with those reported for mycosporine-serine [13,14], for which the exact biosynthetic pathway remains unelucidated. To elucidate the MAAs synthesis pathway in this strain, this study conducted homology-based analysis of proteins using known MAAs biosynthetic enzymes from cyanobacteria as queries. The results of the alignment are presented in Table 2. Homologs for all reference enzymes were successfully identified by aligning the deduced protein sequences from the coding sequence of strain SCSIO-46548, except for Ddl. Due to the presence of functional domains sharing 31% and 38% identity with AroB and DDGS, protein DN4455 matched both proteins. Based on its superior alignment quality with DDGS over O-MT, protein _DN4455 was predicted as the DDGS homolog candidate in SCSIO-46548.

**Figure 7:**
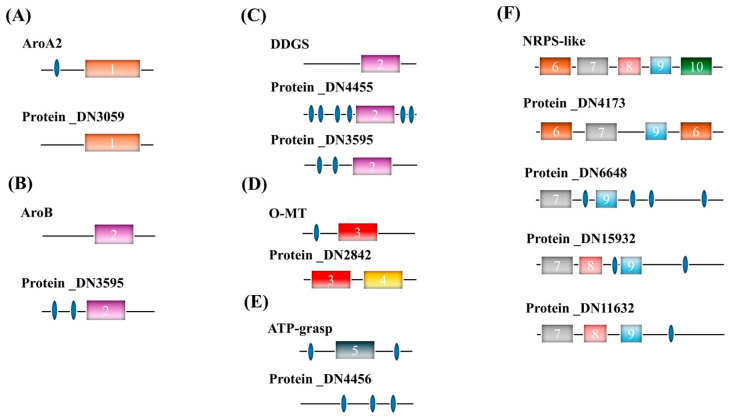
Functional domains of candidate genes involved in MAA biosynthesis in *Asterarcys* sp. SCSIO-46548. (**A**) AroA2; (**B**) AroB; (**C**) DDGS; (**D**) O-MT; (**E**) ATP-grasp; (**F**) NRPS-like. 1, DHAP_synth_2; 2, DHQ_synthase; 3, Methyltransf_3; 4, Adh_short; 5, ATP-grasp_3; 6, Condensation; 7, AMP-binding; 8, AMP-binding_c; 9, PP-bind; 10, Thioesterase. The blue ellipse represents the low-complexity region.

To further confirm the function of the candidate proteins, functional domain prediction and analysis were conducted using the SMART tool, with the results presented in Figure 7. Each candidate protein for AroA2, AroB, DDGS, and O-MT contains its respective key functional domain, including DHAP_synth_2, DHQ_synthase, Methyltransf_3, and ATP-grasp_3, respectively. In contrast, protein _DN4456 lacks the ATP-grasp domain, which may be attributed to the dominant production of mycosporine-serine in SCSIO-46548, a pathway that does not require the glycine-ligation function of ATP-grasp. All four candidate NRPS-like genes contained the essential AMP-binding and PP-binding domains. However, three of them (Protein_DN6648, Protein_DN15932, and Protein_DN11632) displayed negligible expression levels in SCSIO-46548, with TPM values of biosynthetic gene below 1. Therefore, mycosporine-serine might be synthesized via Protein_DN4173 (NRPS-like enzyme) that ligates serine to the 4-DG core structure.

To elucidate how phosphorus limitation promotes MAAs accumulation, the expression of five candidate biosynthetic genes was compared between the phosphorus-limited and control in SCSIO-46548 (Table 3). Under phosphorus limitation, the expression of the gene encoded by Protein_DN4455 (DDGS) was significantly induced, with expression levels elevated by 2.75-fold. Although Protein_DN2842 (O-MT) exhibited a slight decrease in expression level, its basal expression level in SCSIO-46548 remained substantially higher than that of DDGS. Therefore, despite the decrease in O-MT expression, its impact was insufficient to counteract the strong promoting effect of DDGS upregulation. Additionally, the expression level of Protein_DN4173 (NRPS-like) gene showed no significant change.

**Figure 8:**
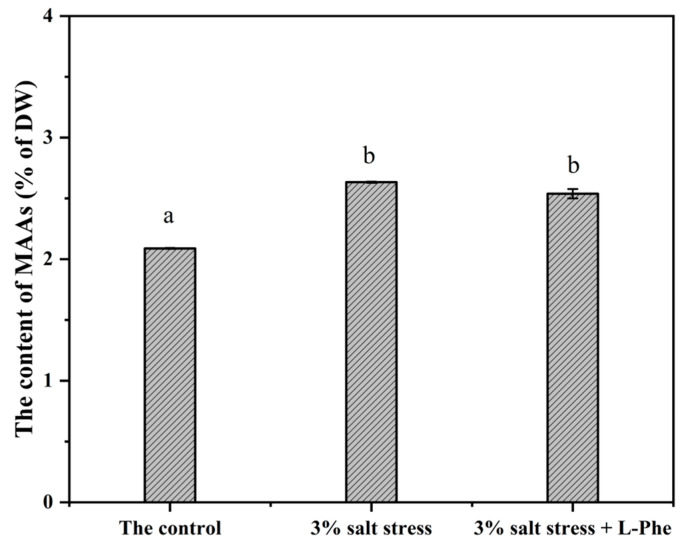
Impact of shikimate pathway inhibition on salt stress-induced MAAs accumulation in *Asterarcys* sp. SCSIO-46548. Different letters denote significant differences among different treatment groups (*p* < 0.05, LSD test).

Moreover, the shikimate pathway genes AroA2 and AroB exhibited a coordinated modest upregulation in response to phosphorus limitation. However, the specific role of this upregulation in MAAs synthesis could not be definitively resolved, as the increased 3-DHQ pool potentially supplies both downstream pathways (Figure 1). To assess the role of the shikimate pathway, salt-stressed cultures of *Asterarcys* sp. SCSIO-46548 were supplemented with the pathway inhibitor L-phenylalanine (L-Phe). As shown in Figure 8, salt stress elevated MAAs synthesis, but this increase was not abolished by co-treatment with the shikimate pathway inhibitor L-phenylalanine. Consequently, in SCSIO-46548, MAAs biosynthesis proceeds predominantly via the pentose phosphate pathway rather than the shikimate pathway. Furthermore, the accumulation of MAAs induced by phosphorus limitation correlated with the upregulation of the DDGS synthesis gene within the pathway.

In the family Scenedesmaceae, several structurally unique MAAs, such as Coelastrin A and Coelastrin B, have been reported [41]. These two compounds are, respectively, a glycine-containing MAA conjugated with γ-dehydrovaline and its 7-O-hexosylated derivative. Nevertheless, the complete biosynthetic pathways of these two compounds remain unclear. Our study on the mycosporine-like serine biosynthetic pathway in Asterarcys sp. SCSIO-46548 may provide clues for their biosynthesis. This could help guide future investigations into the assembly of γ-dehydrovaline adducts and 7-O-hexosylation in Coelastrin A and Coelastrin B.

### 2.6. The Bioinformatic Analysis of Candidate Enzymes in Asterarcys sp. SCSIO-46548

#### 2.6.1. Subcellular Network Prediction

Subcellular localization prediction allows for the decoding of the intrinsic targeting signals within protein sequences, thereby elucidating their potential functions and roles within cellular metabolic networks [42]. In SCSIO-46548, DDGS was predicted to localize to the chloroplasts, which might link its function to light stress response (Table 4). O-MT and NRPS-like were predicted to localize to the cytosol and the plasma membrane, respectively. These results indicated a compartmentalized and sequential organization of the MAAs pathway across distinct subcellular locales in SCSIO-46548. This spatial division could enhance pathway efficiency and ensured targeted delivery of the final photo-protectants to their functional site at the cell periphery.

#### 2.6.2. Physicochemical Characterization

**Figure 9:**
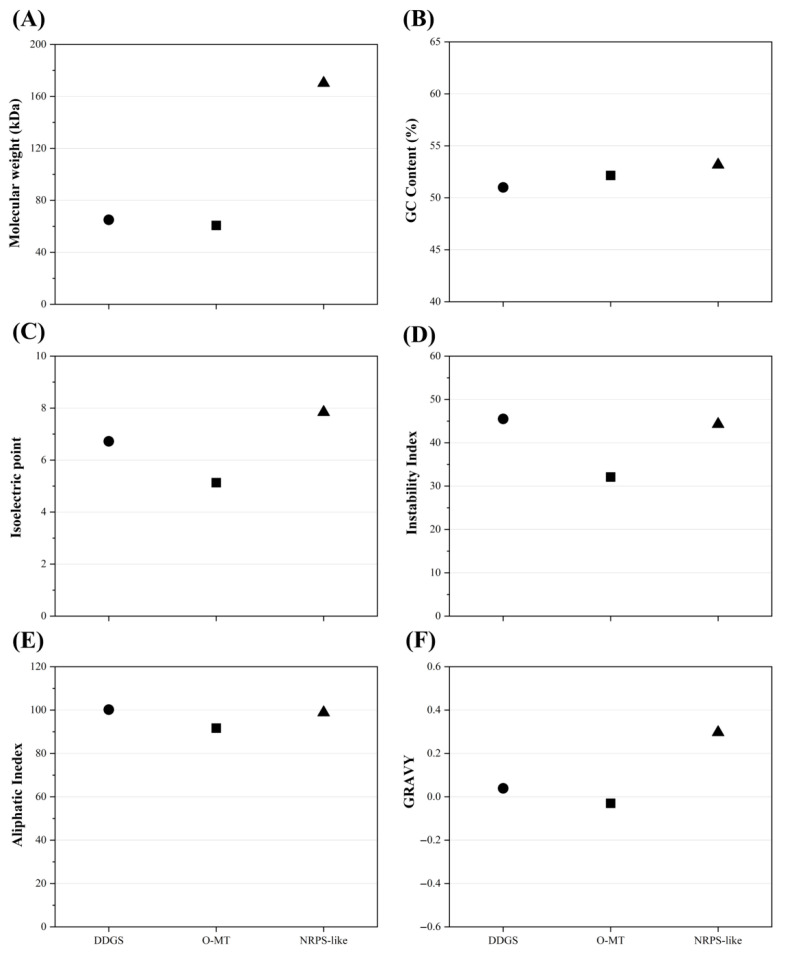
Physicochemical characterization of putative MAAs biosynthetic enzymes and GC content of their encoding genes in *Asterarcys* sp. SCSIO-46548. (**A**) Molecular weights; (**B**) GC contents; (**C**) Isoelectric point; (**D**) Instability index; (**E**) Aliphatic index; (**F**) Grand average of hydropathicity.

The physicochemical characteristics of proteins associated with the MAAs biosynthetic pathway in SCSIO-46548 are presented in Figure 9. The molecular weights of DDGS and O-MT ranged between 60 and 70 kDa, while that of NRPS-like was significantly larger at approximately 170 kDa. This substantial size difference indicated that NRPS-like possessed a longer peptide chain and a more complex structure, which is consistent with the presence of numerous functional domains. The genes for DDGS, O-MT, and NRPS-like showed relatively stable and conserved GC contents, each falling within the range of 50–55%. The isoelectric point (pI) was a fundamental property that dictates the electrostatic behavior, solubility, and optimal purification strategy of a protein [43]. In SCSIO-46548, NRPS-like exhibited a basic isoelectric point (pI > 7), which facilitated its electrostatic interaction with negatively charged phospholipid head groups, thereby enabling stable anchoring to the plasma membrane. In contrast, O-MT had an acidic pI, favoring its solubility and stability within the hydrophilic cytoplasmic environment. The instability index was calculated to be greater than 40 for both DDGS and NRPS-like, but below 40 for O-MT. This indicated that DDGS and NRPS-like are theoretically unstable in vitro and might be more prone to denaturation or degradation under suboptimal conditions. The aliphatic index reflects the relative volume occupied by aliphatic side chains, which is positively correlated with protein thermal stability [44]. The calculated aliphatic indices for DDGS, NRPS-like, and O-MT all fell within the range of 90–100, predicting that these enzymes are thermostable. The grand average of hydropathicity (GRAVY) score provides a quantitative prediction of a protein’s solubility behavior [45]. NRPS-like and DDGS exhibited positive GRAVY values, while O-MT showed a negative value. This indicates that NRPS-like and DDGS are overall hydrophobic in nature, whereas O-MT is hydrophilic. This dichotomy in hydropathic character corresponds to their differential subcellular targeting. Collectively, these findings demonstrate that the key enzymes involved in MAAs biosynthesis in SCSIO-46548 exhibit pronounced functional compartmentalization and robust thermostability. These intrinsic properties suggest considerable potential for their heterologous expression and commercial application.

In summary, phosphorus limitation significantly promotes the accumulation of mycosporine-serine in *Asterarcys* sp. SCSIO-46548, whereas excessive phosphorus deprivation may suppress its accumulation. Notably, phosphorus concentration in the culture medium was varied by changing the amount of K_2_HPO_4_·3H_2_O, which inevitably alters the potassium (K^+^) concentration. However, the impact of reduced K^+^ levels on the observed MAAs accumulation is likely negligible. This is primarily because the concentration of Na^+^ in BG11 medium is approximately 20- to-30-fold higher than that of K^+^. Under such conditions, Na^+^ can substitute for K^+^ in the majority of essential biochemical and physiological processes [46,47], including osmotic balance, charge balance, and activation of certain enzymes.

Regarding the relationship between phosphorus and MAAs accumulation in microalgae, results from different studies are inconsistent. White et al. reported that MAAs content significantly increased under phosphorus limitation in the dinoflagellate *Glenodinium foliaceum* [24]. In contrast, Salehian et al. reported that elevated phosphate levels in the medium promoted MAAs synthesis in the cyanobacterium *Fischerella* sp. F5 [25]. Our findings provide a possible explanation for these discrepancies. In the phosphorus limitation treatment of *Glenodinium foliaceum*, the algal cells may not have experienced severe phosphorus deprivation, resulting in a negative correlation between MAAs content and phosphorus concentration. Conversely, due to the high phosphorus demand of *Fischerella* sp. F5 during growth, phosphorus in the culture medium is rapidly depleted, causing the micoalgal cells to enter a state of phosphate deprivation. This not only severely inhibits its growth but also hinders MAAs accumulation.

These observations provide an effective strategy for the large-scale cultivation of microalgae for MAAs production—appropriately reducing the initial phosphorus concentration in the medium. Nevertheless, the progressive depletion of phosphorus by the algal cells may lead to severe deficiency, posing a limitation on further MAAs biosynthesis. Therefore, real-time fed-batch phosphorus supplementation, based on the photosynthetic and growth status of the microalga, plays a key role in maintaining phosphorus levels and a high rate of MAAs biosynthesis.

In addition to these physiological insights, our transcriptomic analysis provides a molecular foundation for understanding the regulation of MAAs biosynthesis in this strain. Based on the CDS-predicted protein sequences obtained from transcriptomic analysis, we identified the presence of two sets of key enzymes involved in the MAA biosynthetic pathway in *Asterarcys* sp. SCSIO-46548. It was also observed that the pentose phosphate pathway might play a more prominent role in this strain. However, these findings, derived from transcriptomic analysis, require further experimental validation. Future studies should focus on the functional characterization of the identified enzymes through heterologous expression and in vitro enzymatic activity assays.

## 3. Materials and Methods

### 3.1. The Microalga Strain and Maintenance

The microalgal strain was isolated from an outdoor, high-alkaline cyanobacterial (*Plectonema* sp.) culture and identified as *Asterarcys* sp. SCSIO-46548 (PZ251445) based on morphology and 18S rRNA sequencing (Supplementary Materials S1). This strain has been deposited at the South China Sea Institute of Oceanology, Chinese Academy of Sciences (Guangzhou, China), and maintained in the BG11 medium [48], which consisted of 1.5 g/L NaNO_3_, 0.04 g/L K_2_HPO_4_·3H_2_O, 0.036 g/L CaCl_2_, 0.075 g/L MgSO_4_·7H_2_O, 0.006 g/L citric acid, 0.006 g/L (NH_4_)_5_[Fe(C_6_H_4_O_7_)_2_], 0.001 g/L EDTA·2Na, 0.02 g/L Na_2_CO_3_, and 1 mL/L A_5_. The A_5_ solution was made up of the following compositions: 2.86 g/L H_3_BO_3_, 1.81 g/L MnCl_2_·4H_2_O, 0.222 g/L ZnSO_4_·7H_2_O, 0.39 g/L Na_2_MoO_4_·2H_2_O, 0.079 g/L CuSO_4_·5H_2_O, and 0.0494 g/L Co(NO_3_)_2_·6H_2_O.

### 3.2. Experimental Design

#### 3.2.1. The Experiment of Phosphorus Limitation

The culture was carried out in a tubular photobioreactor (Ø6 cm × 60 cm) with an effective culture volume of 1.2 L. And the algal cultures were first pre-cultured in standard BG11 medium to a certain cell density. To establish defined initial phosphorus conditions, algal cultures were centrifuged and washed three times with phosphorus-free BG-11 medium. Following this pretreatment, microalgae cells were inoculated into BG-11 medium containing K_2_HPO_4_·3H_2_O at the specified concentrations (0, 8, 13.3, 20, and 40 mg/L), yielding target initial phosphorus levels of 0, 1.12, 1.87, 2.80, and 5.60 mg/L. Phosphorus concentration in culture medium was varied by changing the amount of K_2_HPO_4_·3H_2_O, without compensating for the concomitant changes in potassium concentration, similar to the experimental design of Hosseinabadi et al. (2022) [49]. The cultivation was conducted under continuous conditions of 26 ± 1 °C, a 24 h photoperiod, and 1% (*v*/*v*) CO_2_ enrichment, with an aeration rate of 0.6 L/min for per photobioreactor. The photon flux density was ramped from 30 to 200 μmol photons/m^2^ s over the first four days and then maintained constant.

#### 3.2.2. Inhibition of the Shikimate Pathway Under Salt Stress

Microalgal cells were cultured in BG11 medium to the logarithmic phase. The control group was continuously cultured in standard BG-11 medium without any additional treatment for the duration of the experiment (72 h). The salt stress group (SS) was subjected directly to 3% (*w*/*v*) NaCl stress for a period of 48 h (F_v_/F_m_ = 0.45, compared to 0.78 in the control). The inhibitor group was first treated with 30 mM L-phenylalanine for 24 h. Subsequently, without changing the medium, NaCl was added to a final concentration of 3% (*w*/*v*) to induce salt stress for an additional 48 h.

### 3.3. Analytial Methods

#### 3.3.1. Determination of Biomass Concentration

A volume of 10 mL of the culture was filtered through a pre-dried filter membrane (0.45 μm) at 80 °C. The filter membrane with microalgae cells was further dried in 80 °C to a constant weight. Biomass concentration was calculated according to the method of Li et al. [50].

#### 3.3.2. The Determination of Photosynthetic Performance

The cultures were diluted to an optical density (OD) of approximately 0.5, measured at 750 nm. The maximum quantum yield of photosystem II (F_v_/F_m_) and non-photochemical quenching (NPQ) were measured using a chlorophyll fluorometer (AP110-C, Photon Systems Instruments, Drásov, Czech Republic) with the following settings [51]. After 30 min of dark adaptation, the initial fluorescence (F_0_) was measured using a weak measuring light (8 μmol photons/m^2^/s). A single saturating pulse (3000 μmol photons/m^2^/s, 0.8 s) was then applied to obtain the maximum fluorescence. Subsequently, the NPQ protocol was initiated. The sample was illuminated with actinic light (200 μmol photons/m^2^/s) for 60 s. During this period, five saturating pulses (3000 μmol photons/m^2^/s, 0.8 s) were applied to measure the light-adapted maximum fluorescence (F_m_′). In the NPQ protocol, the first saturating pulse was applied after 7 s, with subsequent pulses delivered at 12 s intervals.

The photosynthetic efficiency parameters of F_v_/F_m_ and NPQ were calculated via the following formulas [52]:

F_v_/F_m_ = (F_m_ − F_0_)/F_m_ (1)

where F_v_/F_m_ is the maximum photochemical quantum yield of PSII in the dark-adapted state, F_0_ is the initial fluorescence, and F_m_ is the maximum fluorescence in a dark-adapted sample.

NPQ = (F_m_ − F_m_′)/F_m_′ (2)

where NPQ is the non-photochemical quenching coefficient of PSII, F_m_′ is the maximum fluorescence in a light-adapted sample, and F_m_ is the maximum fluorescence in a dark-adapted sample.

#### 3.3.3. Extraction, Purification, Identification, and Quantification of MAAs

Extraction: The extraction procedure of MAAs was optimized as follows: A 4 mL algal culture was harvested, washed three times with deionized water, and extracted with 4 mL of 25% methanol at 48 °C for 15 min with stirring. After cooling, the supernatant was collected by centrifugation (8000 rpm, 5 min). An equal volume of chloroform was added to the supernatant for a 30 min liquid–liquid extraction. The aqueous phase was then filtered through a 0.22 μm aqueous membrane to obtain the MAAs crude extract, whose HPLC chromatogram is shown in Figure S1.

Purification and Identification: The crude extract was passed through a 0.22 μm aqueous membrane filter prior to standard preparation via HPLC. The chromatographic conditions were as follows: YMC-Pack ODS-A semi-preparative column (250 × 10 mm I.D., S-5 μm, 12 nm, YMC Corp., Ltd., Kyoto, Japan); mobile phase consisting of Phase A (HPLC-grade methanol) and Phase B (0.1% acetic acid, HPLC-grade), with isocratic elution at 5% A and 95% B; flow rate of 2.0 mL/min; column temperature of 25 °C. Upon detection of an absorption peak at 320 nm on the monitor, the eluent was collected and subjected to a secondary purification. For the secondary purification, Phase B was replaced with ultrapure water while all other conditions remained unchanged. The absorption peak at 320 nm was collected, freeze-dried, and stored in a freezer at −20 °C. The obtained pure product was then analyzed by HPLC (Figure S2), MS (Figure S3), and MS/MS (Figure S4).

Quantification: Quantification was performed using the standard curve (R^2^ = 0.9994) constructed at 323 nm for the pure product. The content was calculated according to the following:

MAAs content (%) = (C × V)/m × 100%(3)

where C is the MAAs concentration of the crude extract, V is the volume of the extraction reagent, and m is the biomass of the microalgae culture.

#### 3.3.4. The Analysis of Biochemical Composition

Freeze-dried biomass (100 mg DW) was used to determine biochemical composition, including total lipids, lipid classes, total carbohydrates, and proteins. Total lipid content was measured according to Li et al. [53], which is a modified version of the Khozin-Goldberg method [54]. Freeze-dried biomass (10 mg DW) was hydrolyzed with 0.5 N H_2_SO_4_ at 80 °C for 1 h. Total carbohydrate content was then measured using the phenol–sulfuric acid method [55]. For protein analysis, freeze-dried biomass (20 mg DW) was hydrolyzed with 0.5 N NaOH at 80 °C for 20 min, and the procedure was repeated three times. Protein content was determined colorimetrically using the Lowry method [56].

### 3.4. The Comparative Transcriptome Analysis Under Phosphorus Limitation

Total RNA was extracted using a Trizol reagent kit (Shanghai Majorbio Bio-Pharm Technology Co., Ltd.; Shanghai, China) and subsequently used for transcriptome sequencing. The RNA-seq transcriptome library was prepared using the Illumina^®^ Stranded mRNA Prep, Ligation (San Diego, CA, USA) with 1 μg of total RNA. Briefly, mRNA was enriched using oligo(dT) beads via poly(A) selection and then fragmented. Subsequently, double-stranded cDNA was synthesized using random hexamer primers. The synthesized cDNA was then subjected to end repair, phosphorylation, and adapter addition according to the library construction protocol. Libraries were size-selected for cDNA target fragments of 300–400 bp using magnetic beads, followed by PCR amplification for 10–15 cycles. After quantification using a Qubit 4.0 fluorometer (Thermo Fisher Scientific, Waltham, MA, USA), the sequencing library was loaded onto the DNBSEQ-T7 platform (PE150) using the DNBSEQ-T7 RS Reagent Kit (FCL PE150) version 3.0 (MGI Tech, Shenzhen, China). Raw paired-end reads were trimmed and quality-controlled using fastp v1.3.3 with default parameters [57]. Clean data were then subjected to de novo assembly using Trinity v2.15.1 [58]. To improve assembly quality, the assembled sequences were filtered with CD-HIT v4.8.1 [59] and TransRate v1.0.3 [60] and assessed using BUSCO v5.8.2 [61]. The assembled transcripts were aligned to the NCBI NR, COG, and KEGG databases using Diamond v2.1.8 [62] for functional annotation, with an E-value cutoff of 1.0 × 10^−5^. GO annotations were obtained using BLAST2GO v5.2.5 to describe biological processes, molecular functions, and cellular components [63].

Identification of MAAs biosynthetic genes: Putative MAAs biosynthetic enzymes from *Chlorogloeopsis fritschii* were used as queries in BLAST searches [64] against the protein sequences derived from the *Asterarcys* sp. SCSIO-46548 transcriptome. Homologs were identified using stringent thresholds: a bit score ≥ 100 and an E-value ≤ 1.0 × 10^−30^. To identify conserved protein domains, the SMART online tool was employed.

### 3.5. Bioinformatic Analysis of Candidate Enzyme

Subcellular network prediction: To minimize false-positive predictions, the subcellular localization of the target proteins was predicted using a consensus of three complementary bioinformatic tools: TargetP 2.0 [65], CELLO [66], and Cell-PLoc 2.0 [67].

Physicochemical characterization: The molecular weight, theoretical isoelectric point (pI), aliphatic index, and grand average of hydropathicity (GRAVY) of the candidate proteins were predicted using the ExPASy’s ProtParam server (version 3.0). The GC content of the corresponding genes was calculated via the Genscan web service.

### 3.6. Statistical Analysis

Figures show the means and standard deviations of three independent biological replicates and three technical replicates. The statistical analysis (ANOVA) was performed to confirm the differences among treatments using the SPSS 18.0 version. When significant treatment effects were indicated by ANOVA, Fisher’s protected least significant difference (LSD) test was used to compare the individual means at a probability level of 0.05 (e.g., biochemical compositions among different phosphorus limitation groups, and MAAs contents among different treatment groups under salt stress).

## 4. Conclusions

The MAAs biosynthesis machinery in *Asterarcys* sp. SCSIO-46548 comprises both the pentose phosphate pathway and the shikimate pathway, with the pentose phosphate pathway potentially playing a more important role. Phosphorus limitation significantly enhanced the accumulation of MAAs in SCSIO-46548, while severe phosphorus deprivation halted this increase. Further transcriptomic analysis revealed that the induction of MAAs biosynthesis was linked to the upregulation of DDGS expression in the pentose phosphate pathway.

## Figures and Tables

**Table 1:** The expression level of photosynthesis-related proteins in *Asterarcys* sp. SCSIO-46548 under phosphorus limitation.

	Proteins	The Changes in Synthesis
Antenna proteins	LHca2	Down
	LHca4	Down
	LHcb1	Down
	LHcb2	Down
Photosystem II	PsbP	Down
	PsbQ	Down
Photosystem I	PsaG	Down
	PsaO	Down
	PsaN	Down
Photosystem electron transport	PetE	Down
	PetH	Down
	PetJ	Down
F-type ATPase	ATPF1G	Down

“Down” denotes transcripts that were significantly downregulated, with a fold change greater than 2.0.

**Table 2:** BLAST results of known cyanobacterial MAAs biosynthetic enzymes against the protein in *Asterarcys* sp. SCSIO-46548.

Enzymes of MAAs Biosynthesis	Protein ID	BLAST		
		Identity (%)	E-Value	Bit Score
**AroA2**	Protein _DN3059	48	3 × 10^−141^	414
AroB	Protein _DN3595 Protein _DN4455	49 31	3 × 10^−106^ 2 × 10^−32^	318 128
DDGS	Protein _DN4455	38	5 × 10^−79^	255
O-MT	Protein _DN2842	38	7 × 10^−63^	207
ATP-grasp	Protein _DN4456	42	5 × 10^−111^	338
NRPS-like	Protein _DN4173	32	9 × 10^−87^	301
	Protein _DN6648	31	5 × 10^−71^	262
	Protein _DN15932	34	2 × 10^−70^	258
	Protein _DN11632	29	4 × 10^−63^	236

**Table 3:** The expression levels for putative MAAs biosynthetic genes under phosphorus limitation in *Asterarcys* sp. SCSIO-46548.

Enzymes of MAAs Biosynthesis	Protein ID	Expression Level of Gene (TPM)		Ratio
		P-Limitation	The Control	
AroA2	Protein_DN3059	682.84 ± 13.91	477.01 ± 16.47	1.43
AroB	Protein_DN3595	52.22 ± 4.94	35.68 ± 1.34	1.46
DDGS	Protein_DN4455	21.77 ± 3.30	7.91 ± 1.28	2.75
O-MT	Protein_DN2842	91.18 ± 6.10	136.24 ± 1.87	0.67
NRPS-like	Protein_DN4173	33.49 ± 0.03	31.43 ± 2.40	1.07

**Table 4:** Subcellular network prediction of putative MAAs biosynthetic enzymes in *Asterarcys* sp. SCSIO-46548.

Enzymes	Protein ID	Subcellular Network
DDGS	Protein_DN4455	Chloroplast
O-MT	Protein_DN2842	Cytoplasmmic
NRPS-like	Protein_DN4173	Plasma Membrane

## Data Availability

The data presented in this study are available on request from the corresponding author due to legal reasons.

## Supplementary Materials

The following supporting information can be downloaded at: https://www.mdpi.com/article/10.3390/md24050161/s1, S1: The 18S rRNA gene sequence of *Asterarcys* sp. SCSIO-46548; Table S1: Unigene Annotation; Figure S1: HPLC chromatogram of the crude MAAs; Figure S2: HPLC analysis of purified MAAs; Figure S3: MS and spectra of MAAs; Figure S4. MS/MS spectra of MAAs.

## Abbreviations

The following abbreviations are used in this manuscript:

MAAs	Mycosporine-like amino acids
P	Phosphorus
3-DHQ	3-dehydroquinate
DDG	Desmethyl-4-deoxygadusol
4-DG	4-deoxygadusol
DW	Dry weight
DDGS	Desmethyl-4-deoxygadusol synthase
O-MT	O-methyltransferase
NRPS-like	Non-ribosomal peptide synthetase-like enzyme
