# Supplementary material for: Mycosporine-like Amino Acids Biosynthesis in Asterarcys sp. Driving by Phosphorus Limitation: Evidence from Physiological and Transcriptomic Analyses
Source: Mar Drugs. 2026 Apr 30;24(5):161. doi: 10.3390/md24050161 (PMC13208308; doi:10.3390/md24050161)
Supplement: Supplementary file 1 [file marinedrugs-24-00161-s001.zip › marinedrugs-4230273-supplementary.pdf]

SUPPLEMENTARY MATERIALS

Title: Mycosporine-like Amino Acids Biosynthesis in *Asterarcys* sp. Driving by Phosphorus Limitation: Evidence from Physiological and Transcriptomic Analyses

Supplementary materials S1. The 18S rRNA gene sequence of *Asterarcys* sp. SCSIO-46548

GACTGGCCTGGTCACAGATGAAGCCATGCATGTCTAAGTATAAACTGCTTATACT
GTGAAACTGCGAATGGCTCATTAAATCAGTTATAGTTTATTTGGTGGTACCTTACT
ACTCGGATAACCGTAGTAATTCTAGAGCTAATACGTGCGTAAATCCCGACTTCTG
GAAGGGACGTATATATTAGATAAAAGGCCGACCGGGCTTTGCCCGACCCGCGGT
GAATCATGATATCTTCACGAAGCGCATGGCCTTGTGCCGGCGCTGTTCCATTCAA
ATTTCTGCCCTATCAACTTTCGATGGTAGGATAGAGGCCTACCATGGTGGTAACG
GGTGACGGAGGATTAGGGTTCGATTCCGGAGAGGGAGCCTGAGAAACGGCTACC
ACATCCAAGGAAGGCAGCAGGCGCGCAAATTACCCAATCCTGATACGGGGAGG
TAGTGACAATAAATAACAATACCGGGCATTTCATGTCTGGTAATTGGAATGAGTA
CAATCTAAATCCCTTAACGAGGATCCATTGGAGGGCAAGTCTGGTGCCAGCAGC
CGCGGTAATTCCAGCTCCAATAGCGTATATTTAAGTTGTTGCAGTTAAAAAGCTC
GTAGTTGGATTTCGGGTGGGTTCTAGCGGTCCGCCTATGGTGAGTACTGCTATGG
CCTTCCTTTCTGTCGGGGACGGGCTTCTGGGCTTCACTGTCCGGGACTCGGAGTCG
ACGTGGTTACTTTGAGTAAATTAGAGTGTTCAAAGCAGGCTTACGCCCTGAATAC
TTTAGCATGGAATAACACGATAGGACTCTGGCCTATCTTGTTGGTCTGTAGGACT
GGAGTAATGATTAAGAGGGACAGTCGGGGGCATTCGTATTTCATTGTCAGAGGT
GAAATTCTTGGATTTATGAAAGACGAACTACTGCGAAAGCATTTGCCAAGGATG
TTTTCATTAATCAAGAACGAAAGTTGGGGGCTCGAAGACGATTAGATACCGTCGT
AGTCTCAACCATAAACGATGCCGACTAGGGATTGGCGAATGTTTTTTTAATGACT
TCGCCAGCACCTTATGAGAAATCAAAGTTTTTGGGTTCCGGGGGGAGTATGGTCG
CAAGGCTGAAACTTAAAGGAATTGACGGAAGGGCACCACCAGGCGTGGAGCCT
GCGGCTTAATTTGACTCAACACGGGAAAACTTACCAGGTCCAGACATAGTGAGG
ATTGACAGATTGAGAGCTCTTTCTTGATTCTATGGGTGGTGGTGCATGGCCGTTCT
TAGTTGGTGGGTTGCCTTGTCAGGTTGATTCCGGTAACGAACGAGACCTCAGCCT
GCTAAATAGTCCTAGTTGCTTTTTGCAGCTAGCTGACTTCTTAGAGGGACTATTGG
CGTTTAGTCAATGGAAGTATGAGGCAATAACAGGTCTGTGATGCCCTTAGATGTT
CTGGGCCGCACGCGCGCTACACTGATGCATTCAACAAGCCTATCCTTGACCGAA
AGGTCCGGGTAATCTTTGAAACTGCATCGTGATGGGGATAGATTATTGCAATTAT
TAGTCTTCAACGAGGAATGCCTAGTAAGCGCAAGTCATCAGCTTGCGTTGATTAC
GTCCCTGCCCTTTGTACACACCGCCCGTCGCTCCTACCGATTGGGTGTGCTGGTGA
AGTGTTCGGATTGGCAGCTTAGGGTGGCAACACCTCAGGTCTGCCGAGAAGTTCA
TTAACC

TABLE CAPTIONS

Table S1. Unigene Annotation.

FIGURE CAPTIONS

Figure S1. HPLC chromatogram of the crude MAAs.

Figure S2. HPLC analysis of pure MAAs.

Figure S3. MS and spectra of MAAs

Figure S4. MS/MS spectra of MAAs

Table S1. Unigene Annotation.

Sequence File	Matched unigene	Proportion
Total	38733	100%
Total_anno	18749	48.41%
NR	17555	45.32%
KEGG	8414	21.72%
eggNOG	12180	31.45%
GO	14401	37.18%
Swiss-Prot	10453	26.99%
Pfam	12674	32.72%

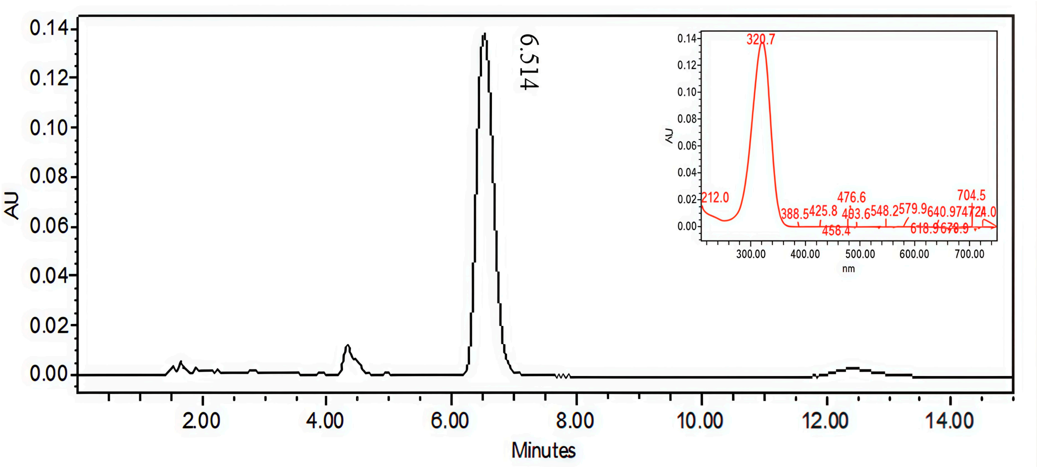

Figure S1. HPLC chromatogram of the crude MAAs

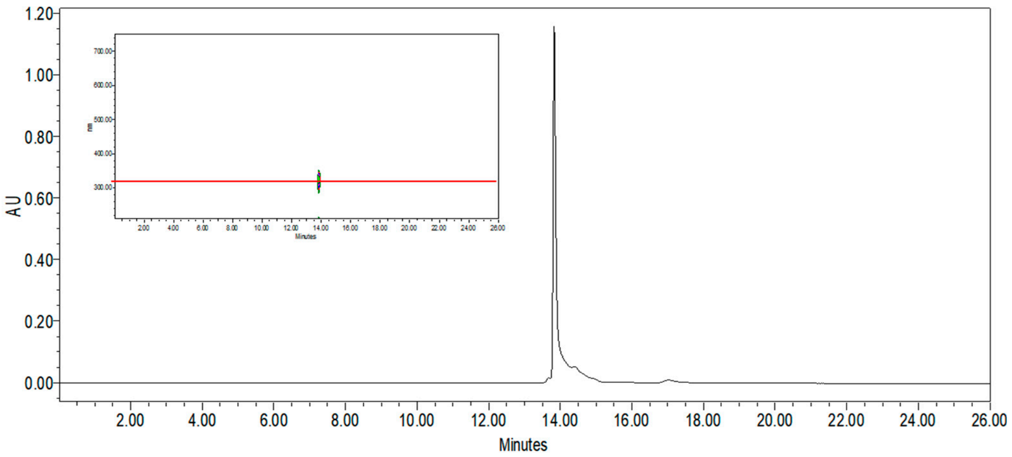

Figure S2. HPLC analysis of purified MAAs

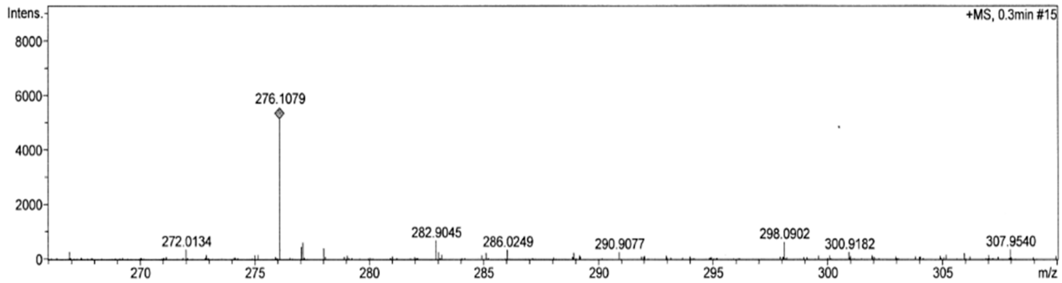

Figure S3. MS and spectra of MAAs

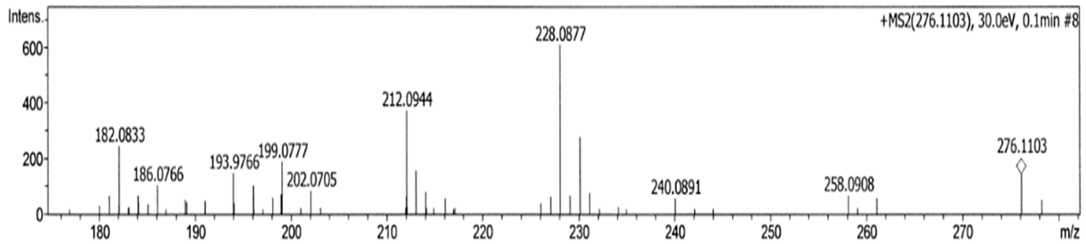

Figure S4. MS/MS spectra of MAAs
